# Essential Oil Chemical Variability in *Oliveria decumbens* (Apiaceae) from Different Regions of Iran and Its Relationship with Environmental Factors

**DOI:** 10.3390/plants9060680

**Published:** 2020-05-27

**Authors:** Akbar Karami, Tahereh Khoshbakht, Hassan Esmaeili, Filippo Maggi

**Affiliations:** 1 Department of Horticultural Science, Faculty of Agriculture, Shiraz University, Shiraz 71441-65186, Iran; akarami2004@gmail.com (A.K.); t.khoshbakht93@gmail.com (T.K.); h.esmaili_6007@yahoo.com (H.E.); 2 School of Pharmacy, University of Camerino, 62032 Camerino, Italy

**Keywords:** essential oil (EO), *Oliveria decumbens*, carvacrol, thymol, environmental factors

## Abstract

*Oliveria decumbens* Vent. (Apiaceae) is an annual herb resistant to harsh environmental conditions, which has got numerous pharmacological, food and feed, and cosmetic applications. In the present study, the variation in the essential oil (EO) content and composition of twelve *O. decumbens* populations growing wild in several habitats of Iran was studied. The EO contents varied from 2.71% (Darab) to 8.52% (Behbahan) on a dry matter basis, where the latter population revealed to be the highest source of essential oil reported so far in this species. Gas chromatography (GC-FID) and gas chromatography-mass spectrometry (GC-MS) analysis revealed that carvacrol (18.8–51.8%), thymol (20.3–38.7%), *γ*-terpinene (0.9–28.8%), *p*-cymene (1.6–21.3%) and myristicin (0.8–9.9%) were the major volatile compounds in all the investigated populations. The EO content had a strong and significant positive correlation with temperature (*r* = 0.62) and sand content (*r* = 0.73), but a strong and significant negative correlation with altitude (*r* = −0.61). On the other hand, the rising altitude led to an increase in thymol content. Cluster and principal component analyses placed the samples from different regions into two main groups based on the main EO components, including thymol/carvacrol type and *γ*-terpinene/thymol/carvacrol/*p*-cymene type. This study provides valuable information for identifying chemotypes in *O. decumbens* as well as insight into planning a domestication and cultivation program.

## 1. Introduction

*Oliveria decumbens* Vent. (Apiaceae) is an annual herb resistant to harsh environmental conditions growing wild in South-East Anatolia, Syria, Iraq and restricted regions of Iran [1]. It is the only species in the *Oliveria* genus which is distributed in subtropical regions of Southwestern Iran including Fars, Kohgiluyeh and Boyer-ahmad, Khuzestan, Kermanshah and Ilam provinces. The plant is also called “Moshkorak”, “Den” or “Denak” by local people [1,2]. The plant flowering begins in early June and proceeds with three color phases, i.e., green, pink-purple and white. The highest content of essential oil (EO) and its major components has been reported in the pink-purple stage [3]. This plant is used in many applications in the traditional Iranian medicine as liver and heart tonic, ant-diarrheal, febrifuge and digestive. Moreover, its antibacterial, antioxidant, anti-*Helicobacter pylori*, anti-cholinesterase, anti-tumour and insecticidal activities have been proven [3,4,5,6,7,8]. The remedial properties of this plant can chiefly be ascribed to its volatile components such as carvacrol and thymol. Previously, thymol, carvacrol, *γ*-terpinene, *p*-cymene and myristicin were identified as the major constituents in the EO of *O. decumbens* from Southern Iran [3,4,9]. Thymol and carvacrol are the most important components of *O. decumbens* EO; they are commonly found in several members of the Lamiaceae, Ranunculaceae, Apiaceae and Verbenaceae families. Numerous biological activities of these phenolic monoterpenes such as antioxidant, antiseptic, antibacterial, antifungal, antiviral, insecticidal, acaricidal, antiprotozoal, antispasmodic, growth promoter, anti-inflammatory and antitussive have been documented [10,11,12,13,14,15,16,17,18]. These numerous properties of its main volatile compounds make this plant a valuable herb for cosmetic, food and feed, and pharmaceutical industries.

Variation of biological properties of EOs is related to variability of their chemical composition, which is in turn influenced by genetic and environmental factors [19,20]. The combination of numerous compounds in complex mixtures such as EOs is determined by genetics but may change under various environmental conditions [21]. The most important ecological factors affecting the quantitative and qualitative value of EO compositions can be the climatic conditions (e.g., light, precipitation, temperature), soil properties (e.g., acidity, soil texture and nutrients) and geographical factors (e.g., latitude and longitude, altitude) [22,23].

Although some studies have been published on the *O. decumbens* EO composition [2,3,4,5,6,7,9,24] to our knowledge a comprehensive analysis focusing on the relationship between the environmental and climatic factors and the chemical compositions have never been provided. Hence, in order to understand the effect of climatic and soil factors on the composition and content of *O. decumbens* EO, twelve naturally growing populations were gathered from different habitats of Iran and analysed by gas chromatography (GC-FID) and by gas chromatography coupled with mass spectrometry (GC-MS). Data analysis, including Pearson’s Correlation Coefficient, Cluster Analysis and Principal Component Analysis, were used to understand the relationships between the chemical compositions and ecological factors. 

## 2. Results and Discussion

### 2.1. Essential Oil Content

**Figure 1:**
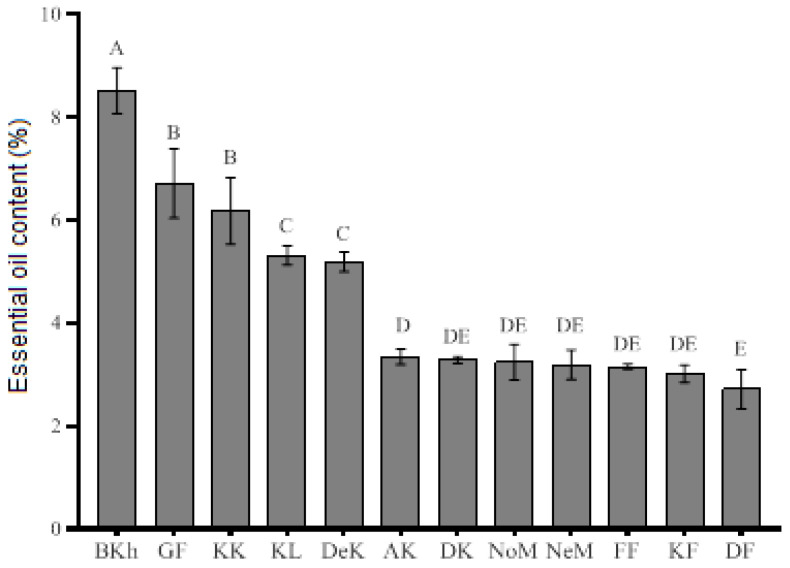
Essential oil (EO) content (%) of different populations of *Oliveria decumbens*. Twelve populations are represented as: Kahnoyeh, Lar, Fars (KL); Darab, Fars (DF); Jahrom, Fars (GF); Farrashband, Fars (FF); Kazeroun, Fars (KF); Konar Takhteh, Kazeroun, Fars (KK); Abolhayat, Kazeroun, Fars (AK); Davan, Kazeroun, Fars (DK); Mamasani Nourabad, Fars (NoM); Neza, Mamasani Nourabad, Fars (NeM); Dehdasht, Kohgiluyeh and Boyer-Ahmad (DeK); Behbahan, Khozestan (BKH).

The EO content in the *O. decumbens* populations collected from different regions varied from 2.71 to 8.52% (Figure 1). The highest and remarkable EO content detected in the population from Behbahan (8.52%) was significantly higher than those previously reported for other areas in Iran and elsewhere. After the Behbahan population, the Jahrom and Konar Takhteh ones showed the highest amount of EO with values equal to 6.58 and 6.17%, respectively. The lowest EO content was related to the population from Darab (2.71%). A previous study on *O. decumbens* revealed that the highest percentage of EO content was 6%, obtained from a population collected in the south of Shiraz [4]. No significant variation was recorded among Darab, Kazeroun, Farashband, Neza, Nourabad and Davan samples.

### 2.2. Volatile Oil Composition

All volatile compounds of EOs, except those occurring in traces, are shown in Table 1. In total, 48 compounds were identified in *O. decumbens* EOs, with carvacrol (18.8–51.8%), thymol (20.3–38.7%), *γ*-terpinene (0.9–28.8%), *p*-cymene (1.6–21.3%) and myristicin (0.8–9.9%) as the main constituents. However, they showed a significant variation related to the sample geographic origin. Among the main compounds mentioned, the highest and lowest coefficient of variation was related to myristicin (62.5%) and thymol (21.7%) (Table 1). The highest carvacrol content was found in the Darab population (51.80%) and this result was significantly different from those reported in previous studies. Additionally, the lowest carvacrol content was detected in the Kahnoyeh population (18.8%). The highest thymol content was found in the Devan (38.7%), Darab (38.6%), Nourabad (36.4%), Neza (35.5%) and Dehdasht (35.2%) populations, whereas the lowest one was recorded in the Kahnoyeh sample (20.3%). The highest (28.8%) and lowest (0.9%) *γ*-terpinene content was found in the Jahrom and Darab population, respectively. The Farashband sample displayed the highest *p*-cymene content (21.3%) whereas that of Darab was the lowest one (1.6%); they showed a significant difference with respect to the other regions. The highest content of myristicin was observed in the Kahnoyeh (9.9%), Abolhayat (9.7%), Dehdasht (9.3%) and Konartakhteh (9.1%) populations, whereas the lowest values were obtained in the Darab (0.8%) and Kazeroun (0.8%) samples. Table 2 summarizes previous studies highlighting quantitative and qualitative changes in the EO chemical composition of *O. decumbens* collected from different regions of Iran [2,3,4,24,25,26,27,28].

### 2.3. Cluster and Principal Component Analysis (PCA)

**Figure 2:**
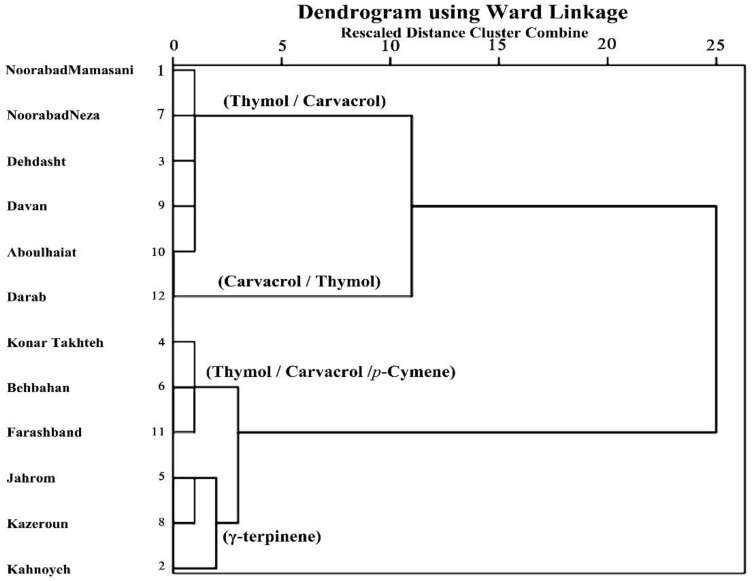
Cluster analysis of the phytochemical composition of *Oliveria decumbens* EO samples from different regions.

The results of cluster analysis showed that the *O. decumbens* EO samples from different regions were classified into two groups based on the main components, including thymol/carvacrol type, and *γ*-terpinene/thymol/carvacrol/*p*-cymene type (Figure 2). The first group includes specimens collected from Nourabad, Neza, Dehdasht, Davan, Abolhayat and Darab, which have a high percentage of thymol and carvacrol. This group can be divided into two subgroups. The first subgroup shows a higher content of thymol (33.3–38.7%) than carvacrol (26.7–33.1%) and includes populations from Nourabad, Neza, Dehdasht, Davan and Abolhayat regions, whereas the second subgroup encompasses only the population from the Darab region showing a higher amount of carvacrol (51.8%) than thymol (38.6%). Therefore, in the first subgroup of the first cluster, the ratio of thymol to carvacrol was more than 1, while in the second subgroup of the first cluster, the ratio of carvacrol to thymol was more than 1. The second group encompassed samples collected from Konartakhteh, Behbahan, Farashband, Jahrom, Kazeroun and Kahnoyeh. The dominant compounds in this group were *γ*-terpinene (19.9–28.8%), thymol (20.3–29.9%), carvacrol (18.8–25.4%) and *p*-cymene (11.0–21.3%). This group was also divided into two subgroups. The first subgroup, including Konartakhteh, Behbahan and Farashband samples, showed a high content of thymol (23.2–24.9%), carvacrol (21.1–22.9%) and *p*-cymene (17.3–21.3%). Instead, the high content of *γ*-terpinene (25.9–28.8%) was a hallmark of the second subgroup, which included Jahrom, Kazeroun and Kahnoyeh samples.

**Figure 3:**
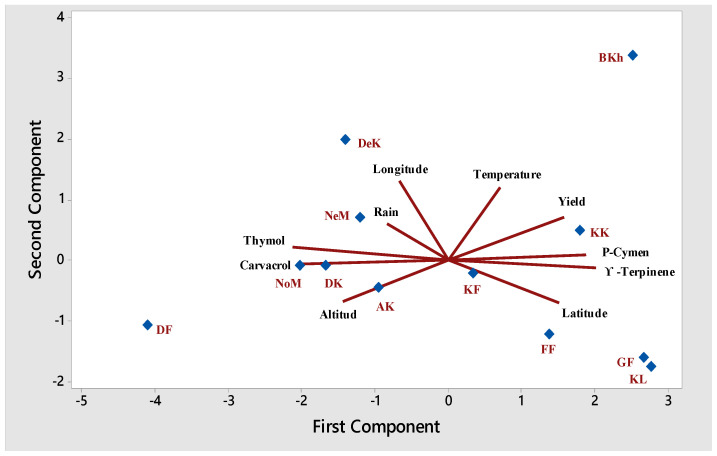
Biplot of the first two principal components (PCs) for the studied populations of *Oliveria decumbens*.

The main EO compounds along with some environmental factors (temperature, altitude, and rain) were selected for principal component analysis (PCA). Cumulative variance obtained for factors are reported in Table 3. PCA relied on three main components explaining 87.3% of the total variance. The results showed that the first principal component (PC1) accounted for 49.6% of the variation in the data and was related to thymol and carvacrol. The second principal component (PC2), explaining 22.5% of variance, was associated with temperature and EO content. The third principal component (PC3) accounted for 15.2% of the variation in the data and was correlated to rain. The results of cluster analysis were confirmed by a biplot based on PCI and PCII in which DF and BKh were placed into two distinct groups. According to the Biplot chart, each of the sampling points identified in the Biplot chart was closer to each of the climatic factor lines, with the closest factor having the greatest influence on the EO of that region (Figure 3).

According to previous studies in *Satureja pilosa* Velen [29] and *Satureja rechingeri* Jamzad [30], the carvacrol, *p*-cymene and thymol content as variables were grouped in the first components of PCA analysis. Similarly, Esmaeili et al. [3] in a previous study on *O. decumbens* showed that thymol and carvacrol contributed to the variance along the PC1.

### 2.4. Correlation between EO Phytochemical Composition and Environmental Factors

Pearson correlation coefficient was performed by SPSS software to calculate the relationship between the phytochemical composition and ecological factors. In the present study, the EO content had a strong and significant positive correlation with environment temperature (*r* = 0.62, significant at the 0.05 level) and sand content (*r* = 0.73, significant at the 0.01 level), but a strong and significant negative correlation with altitude (*r* = −0.61, significant at the 0.05 level) and thymol content (*r* = −0.66, significant at the 0.05 level). In other words, the EO content increased as a function of the temperature and soil sand content rise and decreased with the increase of altitude (data not shown). Similarly, Delazar et al. [31] showed that the medicinal plant *Thymus fedtschenkoi* Ronniger grown at the highest altitude had the lowest content of EO. Consistently with the present results, Jamshidi et al. [32] showed a negative correlation between altitude and the main volatile compound of *T. kotschyanus* Boiss. and Hohen. On the other hand, an inverse correlation between altitude and EO content was found in *Tanacetum polycephalum* Sch.Bip. [33] and *Origanum syriacum* L. [34]. Additionally, phenolic-rich chemotypes in *T. richardii* Pers. were obtained at low altitude and stony soils. Plants grown in areas with cold winters and deep soils showed non-phenolic chemotypes [23,35].

Although the increase in temperature was positively correlated with the increase in the EO content, the effect of this factor on the main EO components was not significant. Novak et al. [36] stated that thymol increased with a reduction of the temperature from 26 to 18 °C while carvacrol accumulated with the increase of temperature from 18 to 26 °C in *Origanum* spp. *p*-Cymene and *γ*-terpinene were negatively correlated with thymol and carvacrol and this confirmed the involvement of these two substances as a precursor in the formation of thymol and carvacrol [37].

In the present study, the thymol content had a significant positive correlation with altitude (*r* = 0.653, significant at the 0.05 level), thus the thymol content increased with the increase of altitude. On the other hand, thymol and γ-terpinene had a significant negative and positive correlation with soil acidity, respectively. Thus, soils with low pH led to a decrease in the thymol content and an increase in the one of *γ*-terpinene. These results are consistent with the ones of Karimi et al. [38] who investigated the ecotypic and chemotypic diversity of *Thymus daenensis* Celak. In their study, they found that altitude had a significant positive effect on the thymol amount and no significant effect on the carvacrol content. In another study conducted on *Satureja thymbra* L. and *Thymbra capitata* (L.) Cav. the maximum carvacrol and thymol content was found in low-altitude and high-altitude lands, respectively [39]. In the study of chemical changes in EOs of *T. praecox* Opiz growing in Southern Turkey, it turned out that although a high amount of thymol was obtained at high altitudes, the EO and carvacrol content decreased with the increase of this factor [40].

Furthermore, the thymol content (*r* = 0.6, significant at the 0.05 level) had a significant and inverse correlation with latitude, whereas carvacrol (*r* = 0.81, significant at the 0.01 level), *p*-cymene (*r* = 0.58, significant at the 0.05 level) and γ-terpinene (*r* = 0.75, significant at the 0.01 level) had a strong and positive correlation with this factor. A study on populations of *Teucrium polium* L. collected from Southern Khuzestan showed that the relative mean of EO compounds varied significantly between populations growing at different latitudes [41].

## 3. Materials and Methods

### 3.1. Collection of Plant and Soil Samples from Natural Habitats

**Figure 4:**
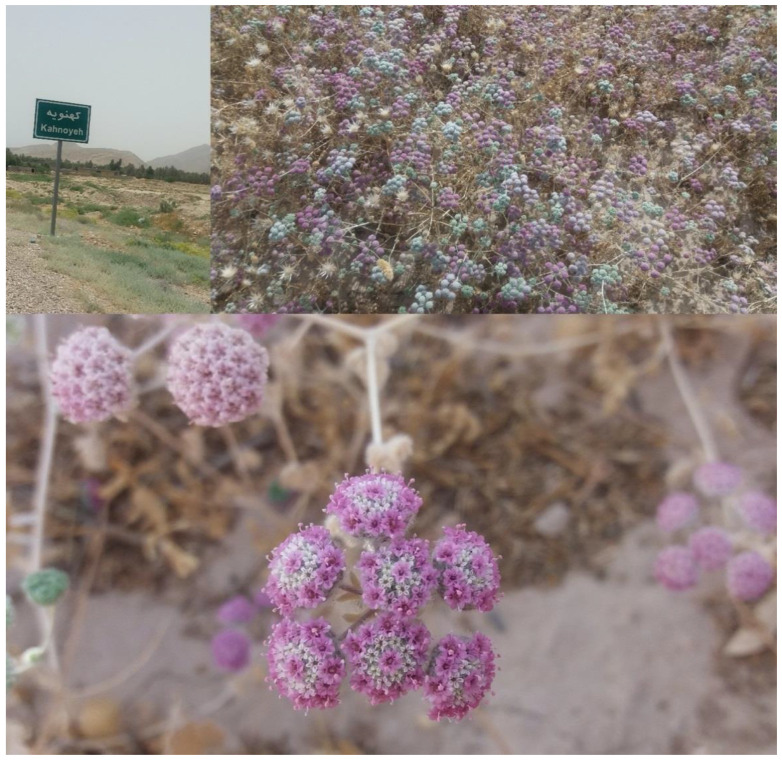
*Oliveria decumbens* at a pink-purple flowering stage from a natural habitat (Kahnoyeh).

**Figure 5:**
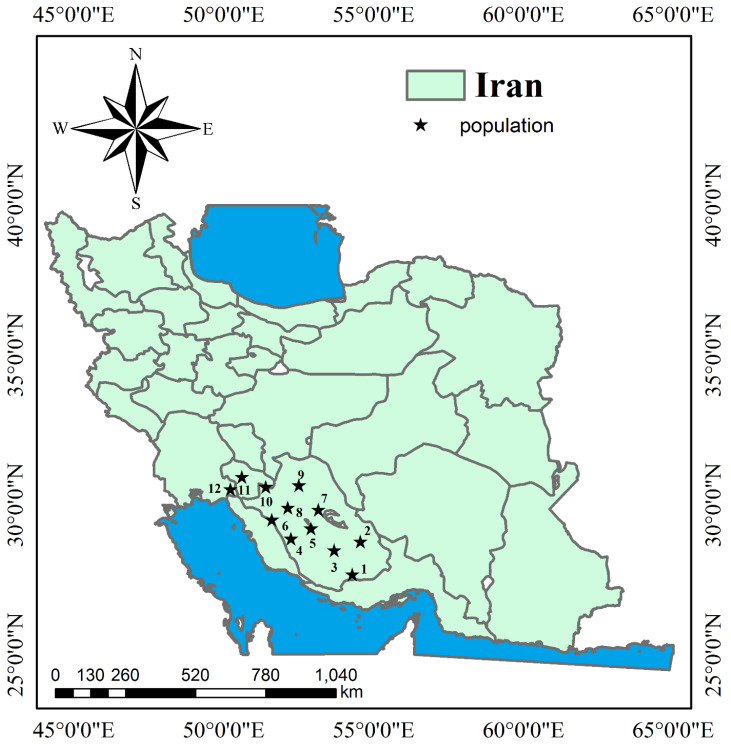
The collected sites for *Oliveria decumbens* across Iran (12 populations are represented by stars). 1: Kahnoyeh, Lar, Fars (KL). 2: Darab, Fars (DF). 3: Jahrom, Fars (GF). 4: Farrashband, Fars (FF). 5: Kazeroun, Fars (KF). 6: Konar Takhteh, Kazeroun, Fars (KK). 7: Abolhayat, Kazeroun, Fars (AK). 8: Davan, Kazeroun, Fars (DK). 9: Mamasani Nourabad, Fars (NoM). 10: Neza, Mamasani Nourabad, Fars (NeM). 11. Dehdasht, Kohgiluyeh and Boyer-Ahmad (DeK). 12. Behbahan, Khozestan (BKH).

The inflorescences of *O. decumbens* Vent. were collected in June 2017 in a phase where the flowers became pink-purple from twelve wild habitats across three provinces of Iran, including Fars (Kazeroun, Davan, Abolhayat, Mamasani, Neza, Kahnoyeh, Konar Takhteh, Jahrom, Farrashband, Darab), Khuzestan (Behbahan) and Kohgiluyeh and Boyer-Ahmad (Dehdasht), as previously described [30] (Figure 4 and Figure 5). Soil samples were taken from a depth of 30 cm to determine their properties. Herbarium specimens were recorded by Dr. Ahmad Reza Khosravi with numbers (55075–55081) and maintained at the Faculty of Science, Department of Biology in Shiraz University. Table 4 shows some climatic and soil characteristics of the sampled populations.

### 3.2. Phytochemical Evaluation

Shade-dried flowers of *O. decumbens* Vent. were used for EO extraction through hydro-distillation by a Clevenger apparatus in three times. 50 g of milled flowers were extracted for 3 h according to the British pharmacopoeia [42] method. The collected EOs were dehydrated by anhydrous sodium sulfate and their content (%, *w/w*) calculated. They were kept at 4 °C under dark conditions until analysis.

A gas chromatograph (GC) Agilent Technologies Model 7890A equipped with a flame ionization detector (FID) and an HP-5 column (30 m × 0.32 mm i.d.; film thickness 0.25 μm) was used. Column thermal programming started at 60 °C, gradually increasing with the rate of 3 °C/min to 210 °C; then the temperature was raised to 240 °C at a rate of 20 °C/min and kept at the final temperature for 8.5 min. The temperature of the injector was 280 °C. Nitrogen was used as the carrier gas with an inlet flow of 1 mL/min.

Gas chromatography-mass spectrometry analysis was performed by the same GC equipped with an Agilent Technologies 5975C mass detector (MS). The column used was a HP-5MS (30 m × 0.32 mm i.d.; film thickness 0.25 μm). The same temperature program of GC-FID was used for analyses. The temperature of the injector and MS detector was 280 °C. The ionization energy was 70 eV. Helium was the carrier gas flowing at 1 mL/min.

The EOs were diluted with dichloromethane and injected into the GC and GC/MS systems and the mass spectra and related chromatograms obtained. The qualitative characteristics of EOs were identified using retention time, retention index, mass spectrum of peaks and comparison with those of available standard compounds or those stored in commercial libraries (WILEY and ADAMS). Semi-quantitative values were determined at GC-FID computing the percentage area of each peak from the total peak area of the chromatogram without using correction factors [43].

### 3.3. Statistical Analyses

Coefficients of variation (CV%) were measured as indicators of variation among all volatile compounds. Cluster analysis, Principal Component Analysis (PCA), and Pearson correlation coefficient were obtained by SPSS software (SPSS Inc., Chicago, IL, USA,) in order to understand the relationship between the phytochemical traits and climatic factors.

## 4. Conclusions

According to the information presented in this study, it can be stated that the quantity and quality of *O. decumbens* EO are affected by environment factors such as altitude, temperature, latitude and soil properties such as soil acidity and sand content. In fact, environmental differences led to the emergence of various chemotypes in this species which can be introduced to the process of domestication and cultivation in order to obtain uniform chemical plants along with appropriate agricultural features. In addition, we were able to introduce an exceptional population with outstanding amount of EO (8.52%). This study has provided the preliminary data on the volatile chemical variability in *O. decumbens*. Therefore, to determine the genetic basis of this diversity and its exploitation in breeding programs, further and broader experiments are needed in the same environment throughout a multi-year sampling.

## Figures and Tables

**Table 1:** Essential oils composition of *Oliveria decumbens* collected from different regions of Iran.

Compound	RI-a	RI-b	Class	KF	DK	AK	NoM	NeM	KL	KK	GF	FF	DF	DeK	BKh	Mean	SD	CV%
α-Thujene	927	930	MH	0.1	0.1	0.1	0.2	0.2	0.3	0.4	0.5	0.2	0.1	0.2	0.4	0.24	0.16	64.7
α-Pinene	934	939	MH	0.1	0.1	0.3	0.1	0.1	0.2	0.2	0.2	0.3	0.3	0	0.2	0.15	0.1	66.6
Camphene	948	954	MH	0	0	0.1	0	0	0.2	0	0	0.4	0	0	0	0.03	0.06	195.4
Sabinene	973	975	MH	0.1	tr	tr	tr	0.1	1.9	0	0	0	0.2	0	0	0.17	0.54	311.2
β–Pinene	978	979	MH	0.9	1.1	1	0.8	1	0	2.5	2.3	1.3	0.4	1.5	2	1.24	0.8	65.1
Myrcene	990	990	MH	0	0	0.3	0	0	0	0	0	1.1	0	0	0	0.12	0.18	248.6
β -Myrcene	991	991	MH	0.5	0.2	0	0.2	0.2	0.5	0.5	0.5	0	0.2	0.2	0.5	0.27	0.21	77.7
*n*-Decane	999	1000	OT	0	0	0	0	0	0	0	0	0	1.2	0	0	0.19	0.66	346.4
α-Phellandrene	1006	1002	MH	0.1	0	0.3	tr	0	tr	tr	0.1	0.2	0	0	tr	0.05	0.09	180.9
α-Terpinene	1018	1017	MH	0.3	0.2	0.6	tr	0.2	0	0.1	0.5	0.5	0	0	0.5	0.25	0.24	97.2
*p*-Cymene	1025	1024	MH	11	10.1	11	9.5	9.7	12.7	18.8	18.3	21.3	1.6	10.1	17.3	12.6	5.6	43.8
Limonene	1028	1029	MH	1.2	0.6	1.3	1.2	1.1	5.5	3.6	1.8	3.1	0.1	1.3	2.5	1.8	1.4	79.1
β–Phellandrene	1030	1030	MH	1.2	0.7	1	0	0	0.4	0	0	0.4	0	0.1	0	0.32	0.44	137
1,8-Cineole	1032	1031	MH	0.1	0.1	0.1	0	0	0	0	0	0.3	0.2	0	0	0.06	0.04	147.7
γ–Terpinene	1060	1059	MH	26.8	11.3	12.9	13	13.9	25.9	19.5	28.8	19.9	0.9	10.6	22.7	16.9	8.2	48.5
Terpinolene	1089	1088	MH	0.1	0.1	0.2	0.1	0.1	tr	0.4	0	Tr	0	0	0.1	0.1	0.11	112.8
Linalool	1099	1096	MO	tr	0.1	tr	tr	0.1	0	0.1	0	0.1	1.4	0	0	0.15	0.4	264.3
α-Terpineol	1190	1188	MO	tr	tr	0.1	0	0.1	tr	tr	0	0.1	0.2	0	0.1	0.04	0.05	123.5
*n*-Dodecane	1198	1200	OT	0	0	0	0	0	0	0	0	0	0.7	0	0	0.05	0.2	346.4
Thymol	1290	1290	MO	29.9	38.7	33.3	36.4	35.5	20.3	23.2	23.1	23.5	38.6	35.2	24.9	30.3	6.6	21.7
Carvacrol	1298	1299	MO	25.4	27.2	26.7	33.1	32.5	18.8	21.1	21.1	22.2	51.8	30.6	22.9	28.03	8.8	31.5
Myristicin	1522	1518	PP	0.8	7	9.7	3.8	4.8	9.9	9.1	2.1	4.3	0.8	9.3	5.3	5.6	3.5	62.5
Elemicin	1577	1557	PP	0	0	tr	0.1	0.1	2.7	0.1	0	0	0	0.6	tr	0.3	0.78	258.1
Total identified (%)				99.3	99.3	99.9	98.5	99.6	97.5	99.8	99.3	99.2	98.7	99.1	99.8	99.7	0.24	

RI-a: Retention indices analyses on HP-5MS column; RI-b: Retention index value taken from ADAMS library; MH: Monoterpene Hydrocarbons; MO: Oxygenated Monoterpenes; PP: Phenylpropanoids; OT: Other compounds; Mean: Means of inter-populations variability; SD: standard deviation; 0: Not detected compounds; tr: Trace (Less than 0.05%).

**Table 2:** Quantitative and qualitative variation of the EO of *Oliveria decumbens* in different regions of Iran (data are taken from previous studies).

Collection Site	Plant Part	EO Content (%)	Thymol (%)	Carvacrol (%)	*γ*-Terpinene (%)	*p*-Cymene (%)	Myristicin (%)	Reference
Kermanshah	Flower	0.1	28	29	20.15	15.4	-	[25]
South of shiraz	Flower	6	47.06	23.31	18.94	8.71	0.36	[4]
Kazeroun	aerial parts	3.4	26.09	0.25	11	13.3	-	[26]
Kuhdasht	aerial parts	1.8	49.9	-	23.1	10	3.2	[27]
Lordegan	aerial parts	2	20.46	9.54	23.32	19.40	21.68	[28]
Kazeroun	aerial parts	6	36.99	17.35	18.94	16.87	0.63	[2]
Bandar abbas	aerial parts	-	34.36	34.8	-	24	20.88	[24]
Kazeroun	aerial parts	2.8	37.8	29.30	10.30	10.07	8.2	[3]

**Table 3:** Eigenvalues and cumulative variance for factors obtained from principal component analysis (PCA) based on major compounds and climatic factors for *Oliveria* *decumbens*.

Variable		Component	
	1	2	3
EO content %	0.320	0.315	0.161
Thymol	−0.428	0.102	−0.143
Carvacrol	−0.402	−0.022	0.325
*p*-Cymene	0.381	0.036	−0.177
γ-Terpinene	0.406	−0.059	−0.134
Altitude	−0.289	−0.305	−0.316
Temperature	0.141	0.533	0.171
Rain	−0.170	0.270	−0.645
Eigenvalue	4.9566	2.2451	1.5244
% of variance	49.6	22.5	15.2
Cumulative %	49.6	72	87.3

**Table 4:** Collection site, soil and geographical characteristics of *Oliveria decumbens* populations.

No	Accession Name	Collection Site	Longitude (E)	Latitude (N)	Altitude (m)	Temp (°C)	Rain (mm)	pH	EC ds/m	Clay (%)	Silt (%)	Sand (%)	N (%)	K (mg/kg)	P (mg/kg)	OM (%)
1	KF	Kazeroun, Fars	51°47’30.6”	29°33’59.04”	950	23.88	27.83	7.26	0.951	12.96	43.83	43.21	0.36	298	53	0.94
2	DK	Davan, Kazeroun, Fars	51°40’41.1”	29°41’51.7”	1445	24.13	28.29	7.15	0.871	20.47	35	44.53	0.15	325	53	0.95
3	AK	Abolhayat, Kazeroun, Fars	51°47’3.84”	29°42’0.16”	1238	23.55	28.04	7.31	1.21	24.64	31	44.36	0.22	365	57	0.92
4	NoM	Mamasani Nourabad, Fars	51°33’16.3”	30°08’21.6”	1268	23.11	35.84	7.57	0.459	11.76	44.72	43.52	0.38	314	75	1.02
5	NeM	Neza, Mamasani Nourabad, Fars	50°53’35.01”	30°04’28.12”	771	23.81	32.21	7.11	0.821	21.76	30	48.24	0.38	354	50	3.23
6	KL	Kahnoyeh, Lar, Fars	53°24’11.8”	27°57’59.6”	758	23.75	18.99	7.59	0.856	19.76	34	46.24	0.14	420	52	0.98
7	KK	Konar Takhteh, Kazeroun, Fars	51°25’14.7”	29°34’41.5”	649	23.56	28.17	7.32	1.05	15.76	36	48.24	0.28	370	66	3.03
8	GF	Jahrom, Fars	53°28’05”	28°33’41”	1108	23.37	21.51	7.5	0.671	23.76	20	56.24	0.13	258	54	0.34
9	FF	Farrashband, Fars	52°0’51.2”	29°01’28.3”	784	23.19	22.17	7.51	0.956	20.26	36	43.74	0.25	416	56	0.94
10	DF	Darab, Fars	48°31’49.5”	28°46’08.7”	1168	23.2	18.55	7.14	0.879	22.67	34	43.33	0.18	362	46	0.86
11	DeK	Dehdasht, Kohgiluyeh and Boyer-Ahmad	50°35’14.5”	30°51’07.3”	971	24.07	34.89	7.18	0.679	23.76	32	44.24	0.11	370	42	0.68
12	BKh	Behbahan, Khozestan	50°23’36.5”	30°32’09.2”	394	25.59	22.20	7.4	0.706	7.76	42	50.24	0.27	210	44	1.08

EC: Electrical conductivity; N: Nitrogen; K: Potassium; P: Phosphor; OM: organic matters; Temp: Mean monthly air temperature; Rain: Mean monthly rain.
